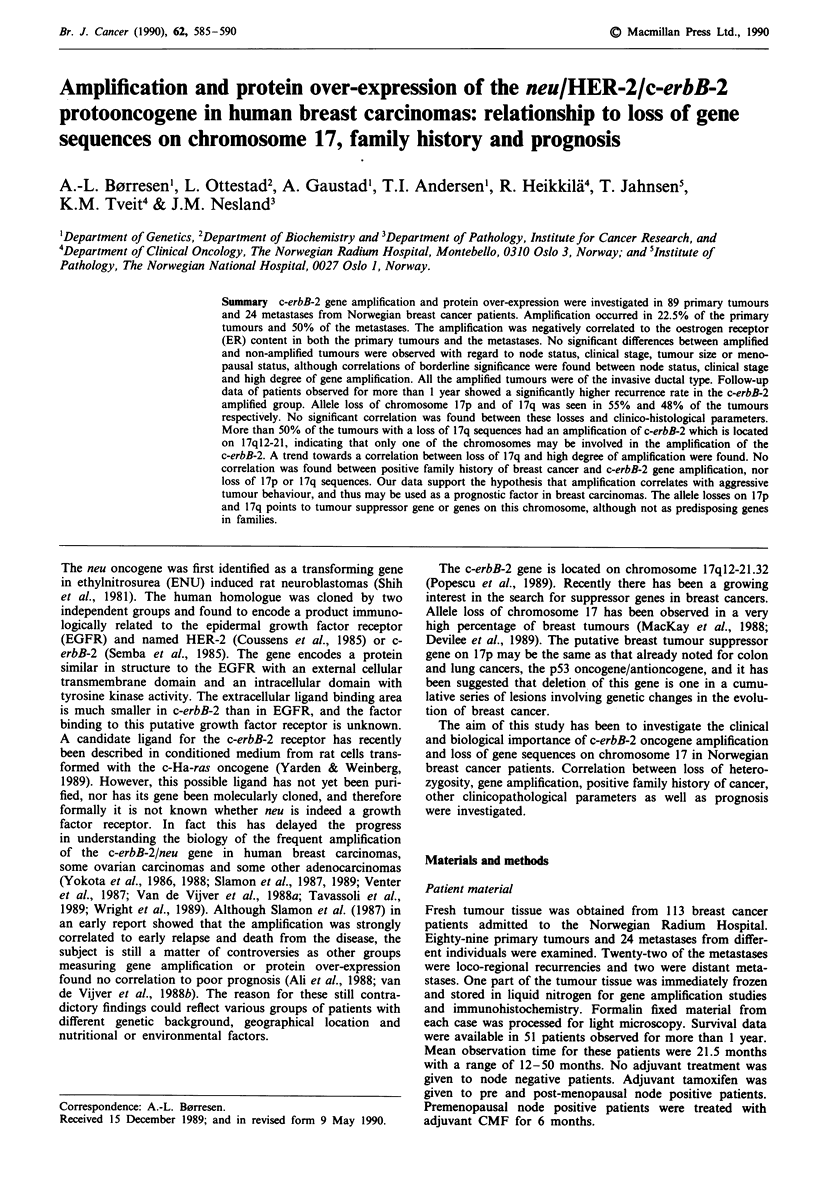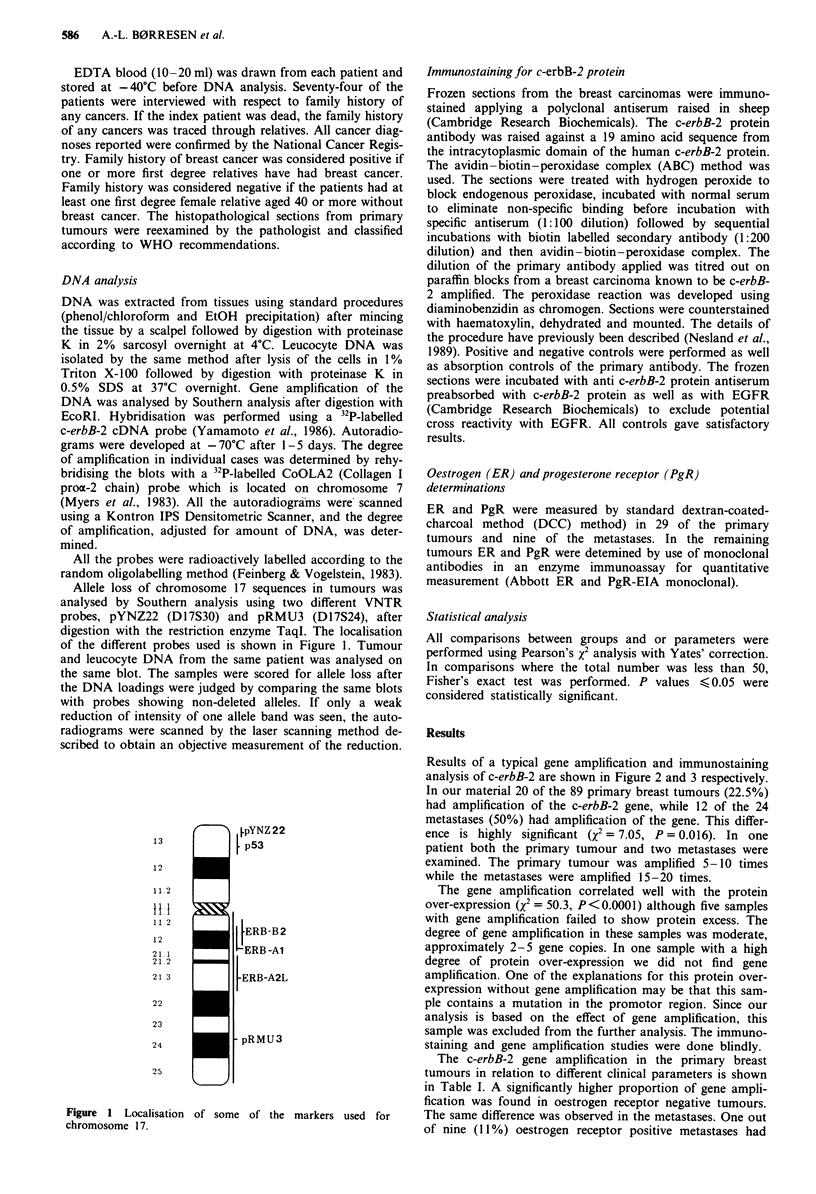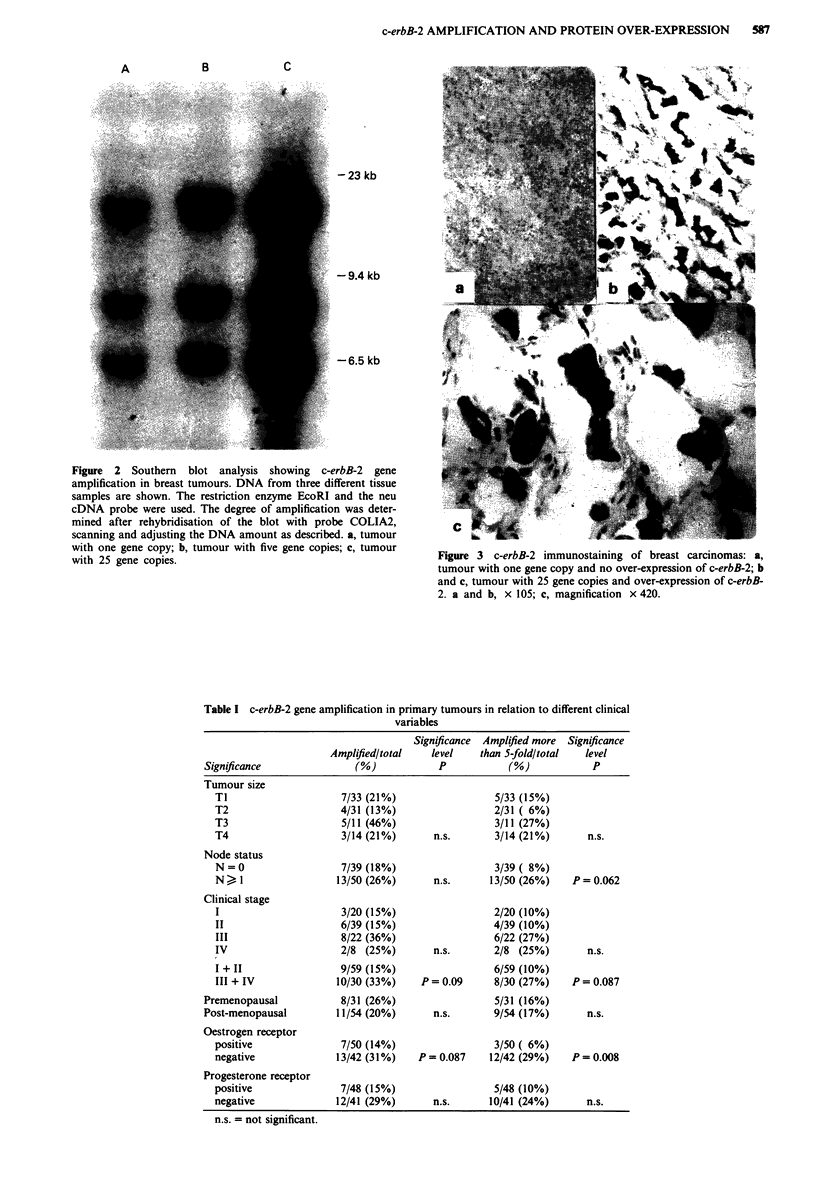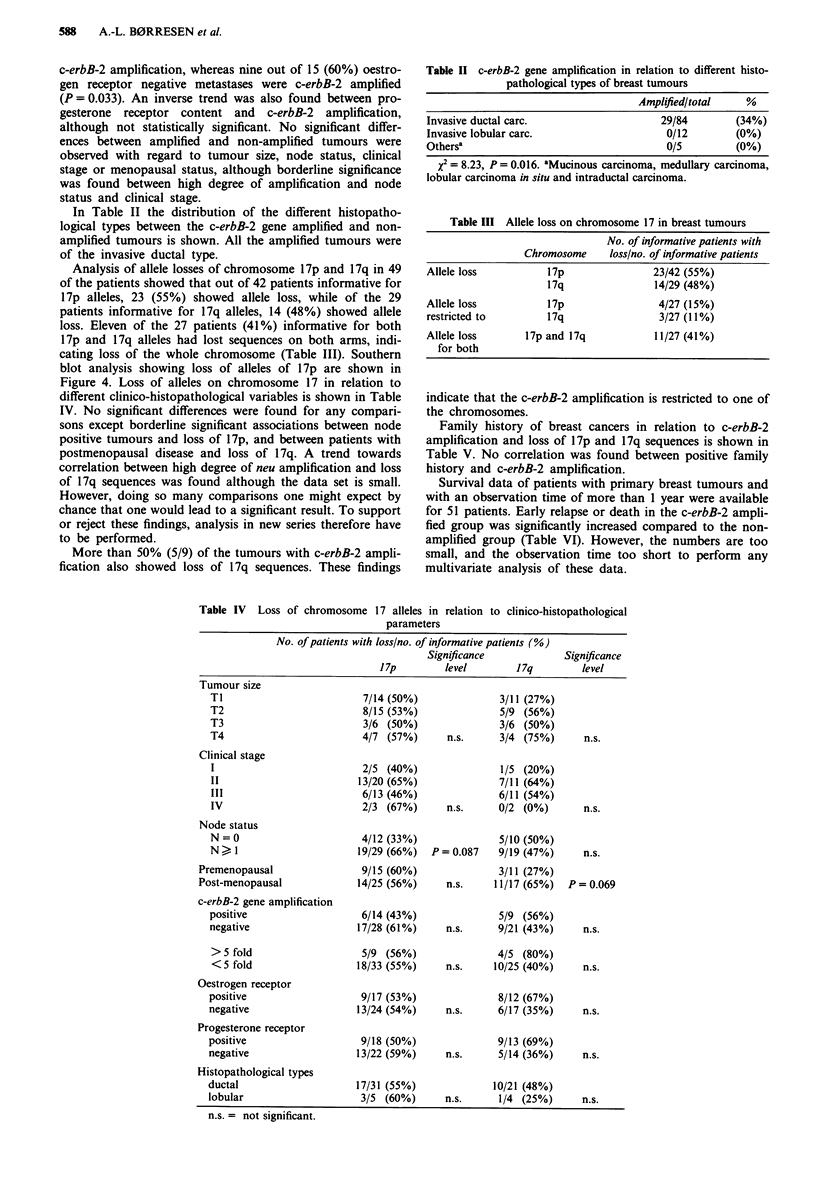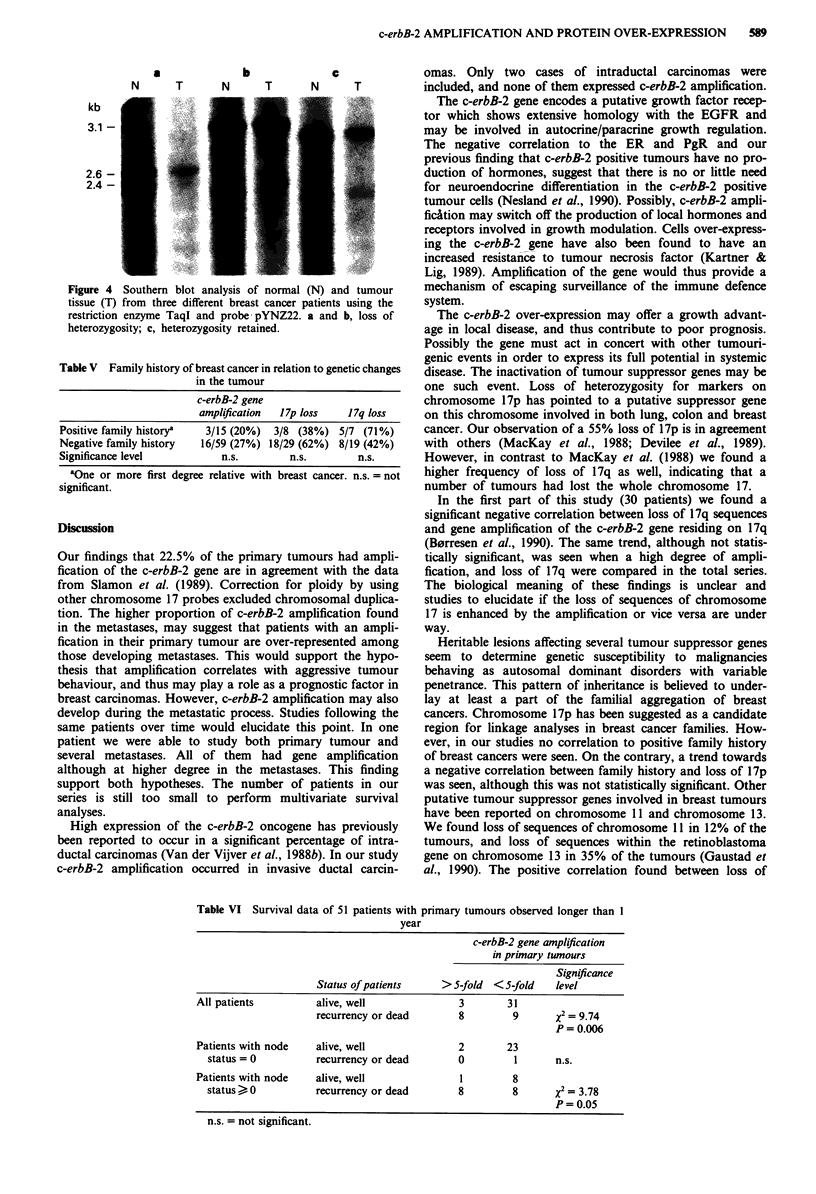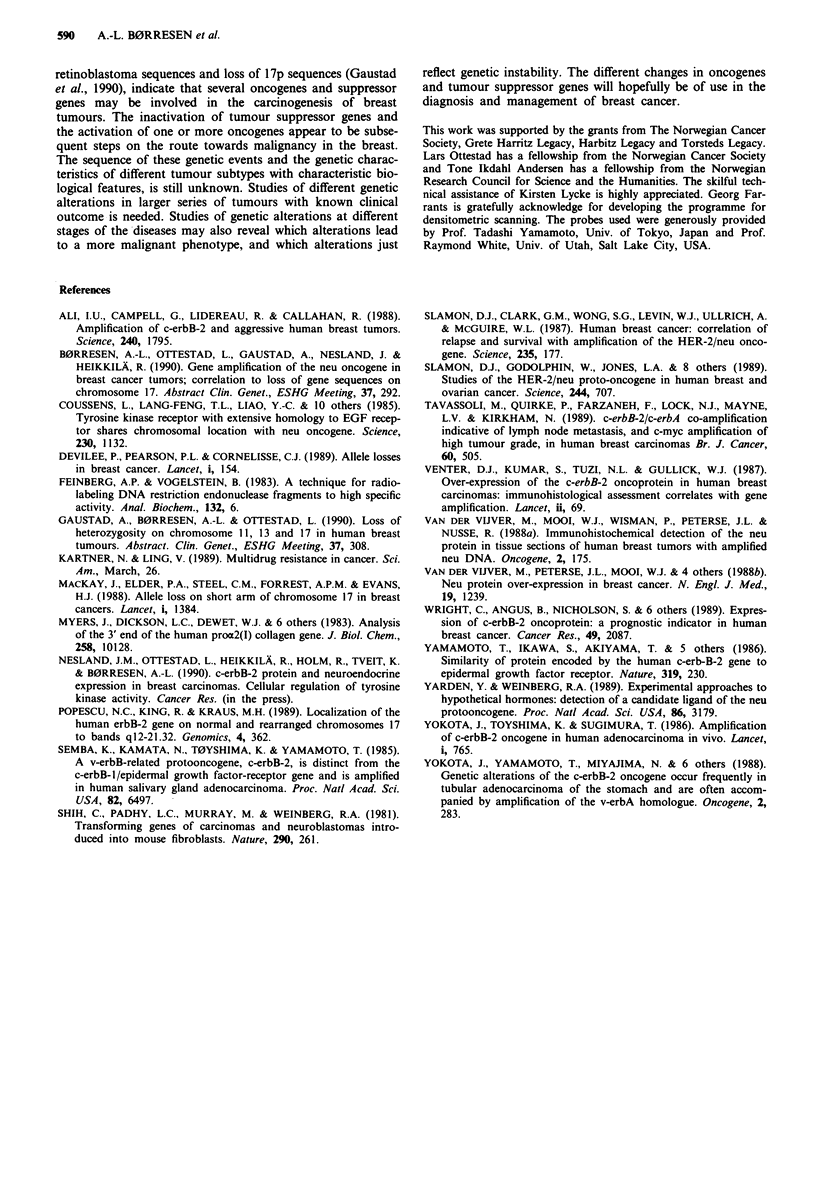# Amplification and protein over-expression of the neu/HER-2/c-erbB-2 protooncogene in human breast carcinomas: relationship to loss of gene sequences on chromosome 17, family history and prognosis.

**DOI:** 10.1038/bjc.1990.334

**Published:** 1990-10

**Authors:** A. L. Børresen, L. Ottestad, A. Gaustad, T. I. Andersen, R. Heikkilä, T. Jahnsen, K. M. Tveit, J. M. Nesland

**Affiliations:** Department of Genetics, Norwegian Radium Hospital, Montebello, Oslo.

## Abstract

**Images:**


Br. J. Cancer (1990), 62, 585–590 © Macmillan Press Ltd., 1990

### Amplification and protein over-expression of the *neu*/HER-2/c-*erbB*-2 protooncogene in human breast carcinomas: relationship to loss of gene sequences on chromosome 17, family history and prognosis

A.-L. Børresen[1], L. Ottestad[2], A. Gaustad[1], T.I. Andersen[1], R. Heikkilä[4], T. Jahnsen[5], K.M. Tveit[4] & J.M. Nesland[3]

[1]*Department of Genetics,* [2]*Department of Biochemistry and* [3]*Department of Pathology, Institute for Cancer Research, and* [4]*Department of Clinical Oncology, The Norwegian Radium Hospital, Montebello, 0310 Oslo 3, Norway; and* [5]*Institute of Pathology, The Norwegian National Hospital, 0027 Oslo 1, Norway.*

**Summary** c-*erbB*-2 gene amplification and protein over-expression were investigated in 89 primary tumours and 24 metastases from Norwegian breast cancer patients. Amplification occurred in 22.5% of the primary tumours and 50% of the metastases. The amplification was negatively correlated to the oestrogen receptor (ER) content in both the primary tumours and the metastases. No significant differences between amplified and non-amplified tumours were observed with regard to node status, clinical stage, tumour size or menopausal status, although correlations of borderline significance were found between node status, clinical stage and high degree of gene amplification. All the amplified tumours were of the invasive ductal type. Follow-up data of patients observed for more than 1 year showed a significantly higher recurrence rate in the c-*erbB*-2 amplified group. Allele loss of chromosome 17p and of 17q was seen in 55% and 48% of the tumours respectively. No significant correlation was found between these losses and clinico-histological parameters. More than 50% of the tumours with a loss of 17q sequences had an amplification of c-*erbB*-2 which is located on 17q12-21, indicating that only one of the chromosomes may be involved in the amplification of the c-*erbB*-2. A trend towards a correlation between loss of 17q and high degree of amplification were found. No correlation was found between positive family history of breast cancer and c-*erbB*-2 gene amplification, nor loss of 17p or 17q sequences. Our data support the hypothesis that amplification correlates with aggressive tumour behaviour, and thus may be used as a prognostic factor in breast carcinomas. The allele losses on 17p and 17q points to tumour suppressor gene or genes on this chromosome, although not as predisposing genes in families.

The *neu* oncogene was first identified as a transforming gene in ethylnitrosurea (ENU) induced rat neuroblastomas (Shih *et al.*, 1981). The human homologue was cloned by two independent groups and found to encode a product immunologically related to the epidermal growth factor receptor (EGFR) and named HER-2 (Coussens *et al.*, 1985) or c-*erbB*-2 (Semba *et al.*, 1985). The gene encodes a protein similar in structure to the EGFR with an external cellular transmembrane domain and an intracellular domain with tyrosine kinase activity. The extracellular ligand binding area is much smaller in c-*erbB*-2 than in EGFR, and the factor binding to this putative growth factor receptor is unknown. A candidate ligand for the c-*erbB*-2 receptor has recently been described in conditioned medium from rat cells transformed with the c-Ha-*ras* oncogene (Yarden & Weinberg, 1989). However, this possible ligand has not yet been purified, nor has its gene been molecularly cloned, and therefore formally it is not known whether *neu* is indeed a growth factor receptor. In fact this has delayed the progress in understanding the biology of the frequent amplification of the c-*erbB*-2/*neu* gene in human breast carcinomas, some ovarian carcinomas and some other adenocarcinomas (Yokota *et al.*, 1986, 1988; Slamon *et al.*, 1987, 1989; Venter *et al.*, 1987; Van de Vijver *et al.*, 1988*a*; Tavassoli *et al.*, 1989; Wright *et al.*, 1989). Although Slamon *et al.* (1987) in an early report showed that the amplification was strongly correlated to early relapse and death from the disease, the subject is still a matter of controversies as other groups measuring gene amplification or protein over-expression found no correlation to poor prognosis (Ali *et al.*, 1988; van de Vijver *et al.*, 1988*b*). The reason for these still contradictory findings could reflect various groups of patients with different genetic background, geographical location and nutritional or environmental factors.

Correspondence: A.-L. Børresen.
Received 15 December 1989; and in revised form 9 May 1990.

The c-*erbB*-2 gene is located on chromosome 17q12-21.32 (Popescu *et al.*, 1989). Recently there has been a growing interest in the search for suppressor genes in breast cancers. Allele loss of chromosome 17 has been observed in a very high percentage of breast tumours (MacKay *et al.*, 1988; Devilee *et al.*, 1989). The putative breast tumour suppressor gene on 17p may be the same as that already noted for colon and lung cancers, the p53 oncogene/antioncogene, and it has been suggested that deletion of this gene is one in a cumulative series of lesions involving genetic changes in the evolution of breast cancer.

The aim of this study has been to investigate the clinical and biological importance of c-*erbB*-2 oncogene amplification and loss of gene sequences on chromosome 17 in Norwegian breast cancer patients. Correlation between loss of heterozygosity, gene amplification, positive family history of cancer, other clinicopathological parameters as well as prognosis were investigated.

### Materials and methods

### Patient material

Fresh tumour tissue was obtained from 113 breast cancer patients admitted to the Norwegian Radium Hospital. Eighty-nine primary tumours and 24 metastases from different individuals were examined. Twenty-two of the metastases were loco-regional recurrencies and two were distant metastases. One part of the tumour tissue was immediately frozen and stored in liquid nitrogen for gene amplification studies and immunohistochemistry. Formalin fixed material from each case was processed for light microscopy. Survival data were available in 51 patients observed for more than 1 year. Mean observation time for these patients were 21.5 months with a range of 12–50 months. No adjuvant treatment was given to node negative patients. Adjuvant tamoxifen was given to pre and post-menopausal node positive patients. Premenopausal node positive patients were treated with adjuvant CMF for 6 months.

EDTA blood (10–20 ml) was drawn from each patient and stored at −40°C before DNA analysis. Seventy-four of the patients were interviewed with respect to family history of any cancers. If the index patient was dead, the family history of any cancers was traced through relatives. All cancer diagnoses reported were confirmed by the National Cancer Registry. Family history of breast cancer was considered positive if one or more first degree relatives have had breast cancer. Family history was considered negative if the patients had at least one first degree female relative aged 40 or more without breast cancer. The histopathological sections from primary tumours were reexamined by the pathologist and classified according to WHO recommendations.

*DNA analysis*

DNA was extracted from tissues using standard procedures (phenol/chloroform and EtOH precipitation) after mincing the tissue by a scalpel followed by digestion with proteinase K in 2% sarcosyl overnight at 4°C. Leucocyte DNA was isolated by the same method after lysis of the cells in 1% Triton X-100 followed by digestion with proteinase K in 0.5% SDS at 37°C overnight. Gene amplification of the DNA was analysed by Southern analysis after digestion with EcoRI. Hybridisation was performed using a $^{32}$P-labelled c-*erbB*-2 cDNA probe (Yamamoto *et al.*, 1986). Autoradiograms were developed at −70°C after 1–5 days. The degree of amplification in individual cases was determined by rehybridising the blots with a $^{32}$P-labelled CoOLA2 (Collagen I pro$\alpha$-2 chain) probe which is located on chromosome 7 (Myers *et al.*, 1983). All the autoradiograms were scanned using a Kontron IPS Densitometric Scanner, and the degree of amplification, adjusted for amount of DNA, was determined.

All the probes were radioactively labelled according to the random oligolabelling method (Feinberg & Vogelstein, 1983).

Allele loss of chromosome 17 sequences in tumours was analysed by Southern analysis using two different VNTR probes, pYNZ22 (D17S30) and pRMU3 (D17S24), after digestion with the restriction enzyme TaqI. The localisation of the different probes used is shown in Figure 1. Tumour and leucocyte DNA from the same patient was analysed on the same blot. The samples were scored for allele loss after the DNA loadings were judged by comparing the same blots with probes showing non-deleted alleles. If only a weak reduction of intensity of one allele band was seen, the autoradiograms were scanned by the laser scanning method described to obtain an objective measurement of the reduction.

**Figure 1** Localisation of some of the markers used for chromosome 17.

*Immunostaining for c-*erbB*-2 protein*

Frozen sections from the breast carcinomas were immunostained applying a polyclonal antiserum raised in sheep (Cambridge Research Biochemicals). The c-*erbB*-2 protein antibody was raised against a 19 amino acid sequence from the intracytoplasmic domain of the human c-*erbB*-2 protein. The avidin–biotin–peroxidase complex (ABC) method was used. The sections were treated with hydrogen peroxide to block endogenous peroxidase, incubated with normal serum to eliminate non-specific binding before incubation with specific antiserum (1:100 dilution) followed by sequential incubations with biotin labelled secondary antibody (1:200 dilution) and then avidin–biotin–peroxidase complex. The dilution of the primary antibody applied was titred out on paraffin blocks from a breast carcinoma known to be c-*erbB*-2 amplified. The peroxidase reaction was developed using diaminobenzidin as chromogen. Sections were counterstained with haematoxylin, dehydrated and mounted. The details of the procedure have previously been described (Nesland *et al.*, 1989). Positive and negative controls were performed as well as absorption controls of the primary antibody. The frozen sections were incubated with anti c-*erbB*-2 protein antiserum preabsorbed with c-*erbB*-2 protein as well as with EGFR (Cambridge Research Biochemicals) to exclude potential cross reactivity with EGFR. All controls gave satisfactory results.

*Oestrogen (ER) and progesterone receptor (PgR) determinations*

ER and PgR were measured by standard dextran-coated-charcoal method (DCC) method) in 29 of the primary tumours and nine of the metastases. In the remaining tumours ER and PgR were detemined by use of monoclonal antibodies in an enzyme immunoassay for quantitative measurement (Abbott ER and PgR-EIA monoclonal).

*Statistical analysis*

All comparisons between groups and or parameters were performed using Pearson's $\chi^2$ analysis with Yates' correction. In comparisons where the total number was less than 50, Fisher's exact test was performed. $P$ values $\leqslant 0.05$ were considered statistically significant.

### Results

Results of a typical gene amplification and immunostaining analysis of c-*erbB*-2 are shown in Figure 2 and 3 respectively. In our material 20 of the 89 primary breast tumours (22.5%) had amplification of the c-*erbB*-2 gene, while 12 of the 24 metastases (50%) had amplification of the gene. This difference is highly significant ($\chi^2 = 7.05$, $P = 0.016$). In one patient both the primary tumour and two metastases were examined. The primary tumour was amplified 5–10 times while the metastases were amplified 15–20 times.

The gene amplification correlated well with the protein over-expression ($\chi^2 = 50.3$, $P < 0.0001$) although five samples with gene amplification failed to show protein excess. The degree of gene amplification in these samples was moderate, approximately 2–5 gene copies. In one sample with a high degree of protein over-expression we did not find gene amplification. One of the explanations for this protein over-expression without gene amplification may be that this sample contains a mutation in the promotor region. Since our analysis is based on the effect of gene amplification, this sample was excluded from the further analysis. The immunostaining and gene amplification studies were done blindly.

The c-*erbB*-2 gene amplification in the primary breast tumours in relation to different clinical parameters is shown in Table I. A significantly higher proportion of gene amplification was found in oestrogen receptor negative tumours. The same difference was observed in the metastases. One out of nine (11%) oestrogen receptor positive metastases had

**Figure 2** Southern blot analysis showing c-*erbB*-2 gene amplification in breast tumours. DNA from three different tissue samples are shown. The restriction enzyme EcoRI and the neu cDNA probe were used. The degree of amplification was determined after rehybridisation of the blot with probe COLIA2, scanning and adjusting the DNA amount as described. **a**, tumour with one gene copy; **b**, tumour with five gene copies; **c**, tumour with 25 gene copies.

**Figure 3** c-*erbB*-2 immunostaining of breast carcinomas: **a**, tumour with one gene copy and no over-expression of c-*erbB*-2; **b** and **c**, tumour with 25 gene copies and over-expression of c-*erbB*-2. **a** and **b**, × 105; **c**, magnification × 420.

**Table I** c-*erbB*-2 gene amplification in primary tumours in relation to different clinical variables

| *Significance* | *Amplified/total (%)* | *Significance level P* | *Amplified more than 5-fold/total (%)* | *Significance level P* |
|---|---|---|---|---|
| Tumour size | | | | |
| T1 | 7/33 (21%) | | 5/33 (15%) | |
| T2 | 4/31 (13%) | | 2/31 ( 6%) | |
| T3 | 5/11 (46%) | | 3/11 (27%) | |
| T4 | 3/14 (21%) | n.s. | 3/14 (21%) | n.s. |
| Node status | | | | |
| N = 0 | 7/39 (18%) | | 3/39 ( 8%) | |
| N ⩾ 1 | 13/50 (26%) | n.s. | 13/50 (26%) | $P = 0.062$ |
| Clinical stage | | | | |
| I | 3/20 (15%) | | 2/20 (10%) | |
| II | 6/39 (15%) | | 4/39 (10%) | |
| III | 8/22 (36%) | | 6/22 (27%) | |
| IV | 2/8 (25%) | n.s. | 2/8 (25%) | n.s. |
| I + II | 9/59 (15%) | | 6/59 (10%) | |
| III + IV | 10/30 (33%) | $P = 0.09$ | 8/30 (27%) | $P = 0.087$ |
| Premenopausal | 8/31 (26%) | | 5/31 (16%) | |
| Post-menopausal | 11/54 (20%) | n.s. | 9/54 (17%) | n.s. |
| Oestrogen receptor | | | | |
| positive | 7/50 (14%) | | 3/50 ( 6%) | |
| negative | 13/42 (31%) | $P = 0.087$ | 12/42 (29%) | $P = 0.008$ |
| Progesterone receptor | | | | |
| positive | 7/48 (15%) | | 5/48 (10%) | |
| negative | 12/41 (29%) | n.s. | 10/41 (24%) | n.s. |

n.s. = not significant.

c-*erbB*-2 amplification, whereas nine out of 15 (60%) oestrogen receptor negative metastases were c-*erbB*-2 amplified ($P = 0.033$). An inverse trend was also found between progesterone receptor content and c-*erbB*-2 amplification, although not statistically significant. No significant differences between amplified and non-amplified tumours were observed with regard to tumour size, node status, clinical stage or menopausal status, although borderline significance was found between high degree of amplification and node status and clinical stage.

In Table II the distribution of the different histopathological types between the c-*erbB*-2 gene amplified and non-amplified tumours is shown. All the amplified tumours were of the invasive ductal type.

Analysis of allele losses of chromosome 17p and 17q in 49 of the patients showed that out of 42 patients informative for 17p alleles, 23 (55%) showed allele loss, while of the 29 patients informative for 17q alleles, 14 (48%) showed allele loss. Eleven of the 27 patients (41%) informative for both 17p and 17q alleles had lost sequences on both arms, indicating loss of the whole chromosome (Table III). Southern blot analysis showing loss of alleles of 17p are shown in Figure 4. Loss of alleles on chromosome 17 in relation to different clinico-histopathological variables is shown in Table IV. No significant differences were found for any comparisons except borderline significant associations between node positive tumours and loss of 17p, and between patients with postmenopausal disease and loss of 17q. A trend towards correlation between high degree of *neu* amplification and loss of 17q sequences was found although the data set is small. However, doing so many comparisons one might expect by chance that one would lead to a significant result. To support or reject these findings, analysis in new series therefore have to be performed.

More than 50% (5/9) of the tumours with c-*erbB*-2 amplification also showed loss of 17q sequences. These findings indicate that the c-*erbB*-2 amplification is restricted to one of the chromosomes.

Family history of breast cancers in relation to c-*erbB*-2 amplification and loss of 17p and 17q sequences is shown in Table V. No correlation was found between positive family history and c-*erbB*-2 amplification.

Survival data of patients with primary breast tumours and with an observation time of more than 1 year were available for 51 patients. Early relapse or death in the c-*erbB*-2 amplified group was significantly increased compared to the non-amplified group (Table VI). However, the numbers are too small, and the observation time too short to perform any multivariate analysis of these data.

**Table II** c-*erbB*-2 gene amplification in relation to different histopathological types of breast tumours

| | *Amplified/total* | % |
|---|---|---|
| Invasive ductal carc. | 29/84 | (34%) |
| Invasive lobular carc. | 0/12 | (0%) |
| Others[a] | 0/5 | (0%) |

$\chi^2 = 8.23$, $P = 0.016$. [a]Mucinous carcinoma, medullary carcinoma, lobular carcinoma *in situ* and intraductal carcinoma.

**Table III** Allele loss on chromosome 17 in breast tumours

| | *Chromosome* | *No. of informative patients with loss/no. of informative patients* |
|---|---|---|
| Allele loss | 17p | 23/42 (55%) |
| | 17q | 14/29 (48%) |
| Allele loss restricted to | 17p | 4/27 (15%) |
| | 17q | 3/27 (11%) |
| Allele loss for both | 17p and 17q | 11/27 (41%) |

**Table IV** Loss of chromosome 17 alleles in relation to clinico-histopathological parameters

| | *No. of patients with loss/no. of informative patients (%)* | | | |
|---|---|---|---|---|
| | *17p* | *Significance level* | *17q* | *Significance level* |
| Tumour size | | | | |
| T1 | 7/14 (50%) | | 3/11 (27%) | |
| T2 | 8/15 (53%) | | 5/9 (56%) | |
| T3 | 3/6 (50%) | | 3/6 (50%) | |
| T4 | 4/7 (57%) | n.s. | 3/4 (75%) | n.s. |
| Clinical stage | | | | |
| I | 2/5 (40%) | | 1/5 (20%) | |
| II | 13/20 (65%) | | 7/11 (64%) | |
| III | 6/13 (46%) | | 6/11 (54%) | |
| IV | 2/3 (67%) | n.s. | 0/2 (0%) | n.s. |
| Node status | | | | |
| $N = 0$ | 4/12 (33%) | | 5/10 (50%) | |
| $N \geqslant 1$ | 19/29 (66%) | $P = 0.087$ | 9/19 (47%) | n.s. |
| Premenopausal | 9/15 (60%) | | 3/11 (27%) | |
| Post-menopausal | 14/25 (56%) | n.s. | 11/17 (65%) | $P = 0.069$ |
| c-*erbB*-2 gene amplification | | | | |
| positive | 6/14 (43%) | | 5/9 (56%) | |
| negative | 17/28 (61%) | n.s. | 9/21 (43%) | n.s. |
| $>5$ fold | 5/9 (56%) | | 4/5 (80%) | |
| $<5$ fold | 18/33 (55%) | n.s. | 10/25 (40%) | n.s. |
| Oestrogen receptor | | | | |
| positive | 9/17 (53%) | | 8/12 (67%) | |
| negative | 13/24 (54%) | n.s. | 6/17 (35%) | n.s. |
| Progesterone receptor | | | | |
| positive | 9/18 (50%) | | 9/13 (69%) | |
| negative | 13/22 (59%) | n.s. | 5/14 (36%) | n.s. |
| Histopathological types | | | | |
| ductal | 17/31 (55%) | | 10/21 (48%) | |
| lobular | 3/5 (60%) | n.s. | 1/4 (25%) | n.s. |

n.s. = not significant.

**Figure 4** Southern blot analysis of normal (N) and tumour tissue (T) from three different breast cancer patients using the restriction enzyme TaqI and probe pYNZ22. **a** and **b**, loss of heterozygosity; **c**, heterozygosity retained.

**Table V** Family history of breast cancer in relation to genetic changes in the tumour

| | *c-erbB-2 gene amplification* | *17p loss* | *17q loss* |
|---|---|---|---|
| Positive family history[a] | 3/15 (20%) | 3/8 (38%) | 5/7 (71%) |
| Negative family history | 16/59 (27%) | 18/29 (62%) | 8/19 (42%) |
| Significance level | n.s. | n.s. | n.s. |

[a]One or more first degree relative with breast cancer. n.s. = not significant.

### Discussion

Our findings that 22.5% of the primary tumours had amplification of the c-*erbB*-2 gene are in agreement with the data from Slamon *et al.* (1989). Correction for ploidy by using other chromosome 17 probes excluded chromosomal duplication. The higher proportion of c-*erbB*-2 amplification found in the metastases, may suggest that patients with an amplification in their primary tumour are over-represented among those developing metastases. This would support the hypothesis that amplification correlates with aggressive tumour behaviour, and thus may play a role as a prognostic factor in breast carcinomas. However, c-*erbB*-2 amplification may also develop during the metastatic process. Studies following the same patients over time would elucidate this point. In one patient we were able to study both primary tumour and several metastases. All of them had gene amplification although at higher degree in the metastases. This finding support both hypotheses. The number of patients in our series is still too small to perform multivariate survival analyses.

High expression of the c-*erbB*-2 oncogene has previously been reported to occur in a significant percentage of intraductal carcinomas (Van der Vijver *et al.*, 1988*b*). In our study c-*erbB*-2 amplification occurred in invasive ductal carcinomas. Only two cases of intraductal carcinomas were included, and none of them expressed c-*erbB*-2 amplification.

The c-*erbB*-2 gene encodes a putative growth factor receptor which shows extensive homology with the EGFR and may be involved in autocrine/paracrine growth regulation. The negative correlation to the ER and PgR and our previous finding that c-*erbB*-2 positive tumours have no production of hormones, suggest that there is no or little need for neuroendocrine differentiation in the c-*erbB*-2 positive tumour cells (Nesland *et al.*, 1990). Possibly, c-*erbB*-2 amplification may switch off the production of local hormones and receptors involved in growth modulation. Cells over-expressing the c-*erbB*-2 gene have also been found to have an increased resistance to tumour necrosis factor (Kartner & Lig, 1989). Amplification of the gene would thus provide a mechanism of escaping surveillance of the immune defence system.

The c-*erbB*-2 over-expression may offer a growth advantage in local disease, and thus contribute to poor prognosis. Possibly the gene must act in concert with other tumourigenic events in order to express its full potential in systemic disease. The inactivation of tumour suppressor genes may be one such event. Loss of heterozygosity for markers on chromosome 17p has pointed to a putative suppressor gene on this chromosome involved in both lung, colon and breast cancer. Our observation of a 55% loss of 17p is in agreement with others (MacKay *et al.*, 1988; Devilee *et al.*, 1989). However, in contrast to MacKay *et al.* (1988) we found a higher frequency of loss of 17q as well, indicating that a number of tumours had lost the whole chromosome 17.

In the first part of this study (30 patients) we found a significant negative correlation between loss of 17q sequences and gene amplification of the c-*erbB*-2 gene residing on 17q (Børresen *et al.*, 1990). The same trend, although not statistically significant, was seen when a high degree of amplification, and loss of 17q were compared in the total series. The biological meaning of these findings is unclear and studies to elucidate if the loss of sequences of chromosome 17 is enhanced by the amplification or vice versa are under way.

Heritable lesions affecting several tumour suppressor genes seem to determine genetic susceptibility to malignancies behaving as autosomal dominant disorders with variable penetrance. This pattern of inheritance is believed to underlay at least a part of the familial aggregation of breast cancers. Chromosome 17p has been suggested as a candidate region for linkage analyses in breast cancer families. However, in our studies no correlation to positive family history of breast cancers were seen. On the contrary, a trend towards a negative correlation between family history and loss of 17p was seen, although this was not statistically significant. Other putative tumour suppressor genes involved in breast tumours have been reported on chromosome 11 and chromosome 13. We found loss of sequences of chromosome 11 in 12% of the tumours, and loss of sequences within the retinoblastoma gene on chromosome 13 in 35% of the tumours (Gaustad *et al.*, 1990). The positive correlation found between loss of

**Table VI** Survival data of 51 patients with primary tumours observed longer than 1 year

| | | *c-erbB-2 gene amplification in primary tumours* | | |
|---|---|---|---|---|
| | *Status of patients* | *> 5-fold* | *< 5-fold* | *Significance level* |
| All patients | alive, well | 3 | 31 | |
| | recurrency or dead | 8 | 9 | $\chi^2 = 9.74$ $P = 0.006$ |
| Patients with node status = 0 | alive, well | 2 | 23 | |
| | recurrency or dead | 0 | 1 | n.s. |
| Patients with node status $\geqslant 0$ | alive, well | 1 | 8 | |
| | recurrency or dead | 8 | 8 | $\chi^2 = 3.78$ $P = 0.05$ |

n.s. = not significant.

retinoblastoma sequences and loss of 17p sequences (Gaustad et al., 1990), indicate that several oncogenes and suppressor genes may be involved in the carcinogenesis of breast tumours. The inactivation of tumour suppressor genes and the activation of one or more oncogenes appear to be subsequent steps on the route towards malignancy in the breast. The sequence of these genetic events and the genetic characteristics of different tumour subtypes with characteristic biological features, is still unknown. Studies of different genetic alterations in larger series of tumours with known clinical outcome is needed. Studies of genetic alterations at different stages of the diseases may also reveal which alterations lead to a more malignant phenotype, and which alterations just reflect genetic instability. The different changes in oncogenes and tumour suppressor genes will hopefully be of use in the diagnosis and management of breast cancer.

This work was supported by the grants from The Norwegian Cancer Society, Grete Harritz Legacy, Harbitz Legacy and Torsteds Legacy. Lars Ottestad has a fellowship from the Norwegian Cancer Society and Tone Ikdahl Andersen has a fellowship from the Norwegian Research Council for Science and the Humanities. The skilful technical assistance of Kirsten Lycke is highly appreciated. Georg Farrants is gratefully acknowledge for developing the programme for densitometric scanning. The probes used were generously provided by Prof. Tadashi Yamamoto, Univ. of Tokyo, Japan and Prof. Raymond White, Univ. of Utah, Salt Lake City, USA.

### References

ALI, I.U., CAMPELL, G., LIDEREAU, R. & CALLAHAN, R. (1988). Amplification of c-erbB-2 and aggressive human breast tumors. *Science*, **240**, 1795.

BØRRESEN, A.-L., OTTESTAD, L., GAUSTAD, A., NESLAND, J. & HEIKKILÄ, R. (1990). Gene amplification of the neu oncogene in breast cancer tumors; correlation to loss of gene sequences on chromosome 17. *Abstract Clin. Genet., ESHG Meeting*, **37**, 292.

COUSSENS, L., LANG-FENG, T.L., LIAO, Y.-C. & 10 others (1985). Tyrosine kinase receptor with extensive homology to EGF receptor shares chromosomal location with neu oncogene. *Science*, **230**, 1132.

DEVILEE, P., PEARSON, P.L. & CORNELISSE, C.J. (1989). Allele losses in breast cancer. *Lancet*, **i**, 154.

FEINBERG, A.P. & VOGELSTEIN, B. (1983). A technique for radiolabeling DNA restriction endonuclease fragments to high specific activity. *Anal. Biochem.*, **132**, 6.

GAUSTAD, A., BØRRESEN, A.-L. & OTTESTAD, L. (1990). Loss of heterozygosity on chromosome 11, 13 and 17 in human breast tumours. *Abstract. Clin. Genet., ESHG Meeting*, **37**, 308.

KARTNER, N. & LING, V. (1989). Multidrug resistance in cancer. *Sci. Am.*, March, 26.

MACKAY, J., ELDER, P.A., STEEL, C.M., FORREST, A.P.M. & EVANS, H.J. (1988). Allele loss on short arm of chromosome 17 in breast cancers. *Lancet*, **i**, 1384.

MYERS, J., DICKSON, L.C., DEWET, W.J. & 6 others (1983). Analysis of the 3′ end of the human proα2(I) collagen gene. *J. Biol. Chem.*, **258**, 10128.

NESLAND, J.M., OTTESTAD, L., HEIKKILÄ, R., HOLM, R., TVEIT, K. & BØRRESEN, A.-L. (1990). c-erbB-2 protein and neuroendocrine expression in breast carcinomas. Cellular regulation of tyrosine kinase activity. *Cancer Res.* (in the press).

POPESCU, N.C., KING, R. & KRAUS, M.H. (1989). Localization of the human erbB-2 gene on normal and rearranged chromosomes 17 to bands q12-21.32. *Genomics*, **4**, 362.

SEMBA, K., KAMATA, N., TØYSHIMA, K. & YAMAMOTO, T. (1985). A v-erbB-related protooncogene, c-erbB-2, is distinct from the c-erbB-1/epidermal growth factor-receptor gene and is amplified in human salivary gland adenocarcinoma. *Proc. Natl Acad. Sci. USA*, **82**, 6497.

SHIH, C., PADHY, L.C., MURRAY, M. & WEINBERG, R.A. (1981). Transforming genes of carcinomas and neuroblastomas introduced into mouse fibroblasts. *Nature*, **290**, 261.

SLAMON, D.J., CLARK, G.M., WONG, S.G., LEVIN, W.J., ULLRICH, A. & MCGUIRE, W.L. (1987). Human breast cancer: correlation of relapse and survival with amplification of the HER-2/neu oncogene. *Science*, **235**, 177.

SLAMON, D.J., GODOLPHIN, W., JONES, L.A. & 8 others (1989). Studies of the HER-2/neu proto-oncogene in human breast and ovarian cancer. *Science*, **244**, 707.

TAVASSOLI, M., QUIRKE, P., FARZANEH, F., LOCK, N.J., MAYNE, L.V. & KIRKHAM, N. (1989). *c-erbB-2/c-erbA* co-amplification indicative of lymph node metastasis, and c-myc amplification of high tumour grade, in human breast carcinomas *Br. J. Cancer*, **60**, 505.

VENTER, D.J., KUMAR, S., TUZI, N.L. & GULLICK, W.J. (1987). Over-expression of the *c-erbB-2* oncoprotein in human breast carcinomas: immunohistological assessment correlates with gene amplification. *Lancet*, **ii**, 69.

VAN DER VIJVER, M., MOOI, W.J., WISMAN, P., PETERSE, J.L. & NUSSE, R. (1988*a*). Immunohistochemical detection of the neu protein in tissue sections of human breast tumors with amplified neu DNA. *Oncogene*, **2**, 175.

VAN DER VIJVER, M., PETERSE, J.L., MOOI, W.J. & 4 others (1988*b*). Neu protein over-expression in breast cancer. *N. Engl. J. Med.*, **19**, 1239.

WRIGHT, C., ANGUS, B., NICHOLSON, S. & 6 others (1989). Expression of c-erbB-2 oncoprotein: a prognostic indicator in human breast cancer. *Cancer Res.*, **49**, 2087.

YAMAMOTO, T., IKAWA, S., AKIYAMA, T. & 5 others (1986). Similarity of protein encoded by the human c-erb-B-2 gene to epidermal growth factor receptor. *Nature*, **319**, 230.

YARDEN, Y. & WEINBERG, R.A. (1989). Experimental approaches to hypothetical hormones: detection of a candidate ligand of the neu protooncogene. *Proc. Natl Acad. Sci. USA*, **86**, 3179.

YOKOTA, J., TOYSHIMA, K. & SUGIMURA, T. (1986). Amplification of c-erbB-2 oncogene in human adenocarcinoma in vivo. *Lancet*, **i**, 765.

YOKOTA, J., YAMAMOTO, T., MIYAJIMA, N. & 6 others (1988). Genetic alterations of the c-erbB-2 oncogene occur frequently in tubular adenocarcinoma of the stomach and are often accompanied by amplification of the v-erbA homologue. *Oncogene*, **2**, 283.